# Infrared Spectroscopy of Au^+^(CH_4_)_*n*_ Complexes and Vibrationally-Enhanced C–H Activation Reactions

**DOI:** 10.1007/s11244-017-0868-z

**Published:** 2017-10-30

**Authors:** Alexander S. Gentleman, Alice E. Green, Daniel R. Price, Ethan M. Cunningham, Andreas Iskra, Stuart R. Mackenzie

**Affiliations:** Physical and Theoretical Chemistry Laboratory, Department of Chemistry, University of Oxford, South Parks Road, Oxford, OX1 3QZ UK

**Keywords:** Infrared photodissociation, Metal complex, Dissociation energy

## Abstract

A combined spectroscopic and computational study of gas-phase Au^+^(CH_4_)_*n*_ (*n* = 3–8) complexes reveals a strongly-bound linear Au^+^(CH_4_)_2_ core structure to which up to four additional ligands bind in a secondary coordination shell. Infrared resonance-enhanced photodissociation spectroscopy in the region of the CH_4_ *a*_1_ and *t*_2_ fundamental transitions reveals essentially free internal rotation of the core ligands about the H_4_C–Au^+^–CH_4_ axis, with sharp spectral features assigned by comparison with spectral simulations based on density functional theory. In separate experiments, vibrationally-enhanced dehydrogenation is observed when the *t*_2_ vibrational normal mode in methane is excited prior to complexation. Clear infrared-induced enhancement is observed in the mass spectrum for peaks corresponding 4u below the mass of the Au^+^(CH_4_)_*n*=2,3_ complexes corresponding, presumably, to the loss of two H_2_ molecules.

## Introduction

Isolated gas-phase metal ion–ligand clusters in many ways represent ideal model systems for studying fundamental metal–ligand interactions. Studies of these systems provide insight into metal–ligand binding energies and weakening of ligand internal bonds while remaining free from complex solvation, counterion, or defect effects. In turn, these shed light on the mechanism by which ligand activation occurs, leading to enhanced understanding of catalytic reactivity. Many industrial heterogeneous catalysts typically comprise multiple components, with reactions assumed to occur at metal defects supported by a macroscopic bulk structure [1]. The model systems studied can capture some of the features and energetics of the surface of catalysts [2], without complications arising from the bulk structure. The simplified nature of these model complexes also makes them tractable to theoretical studies which, coupled with experimental data, can provide a deeper understanding of fundamental interactions involved. These studies are often too computationally-intensive to perform rigorously on real systems.

The interaction and reaction of methane with transition metal ions is of particular practical interest. As the simplest saturated hydrocarbon, methane serves as a key model for understanding metal ion–hydrocarbon systems. The activation of methane is an intensively studied topic in catalysis [3–5]. Methane is the primary component in natural gas (typically 80–90%—[4]) and with depleting petroleum reserves, the possibility of easily converting methane to more valuable chemicals and fuels would lead to its use as an abundant hydrocarbon feedstock.

For complete methane activation to occur, at least one of the four strong C–H bonds (bond dissociation energy of 439 kJ mol^−1^—[6]) must undergo bond scission. This is made challenging by the paucity of low-energy empty and high-energy filled orbitals in methane, making it relatively inert to reaction under most conditions [3]. The classic industrial route used to convert methane into useful reagents involves the initial conversion of methane to syngas (CO + H_2_) *via* steam reforming, followed by the conversion of this syngas into a range of hydrocarbons or alcohols [3–5]. Despite representing a useful H_2_ source, the steam reforming of methane is energy-intensive, requiring high temperatures and consequent high capital costs associated with industrial operations [3, 4].

Many studies have investigated bulk-phase catalysts and reaction environments for directly converting methane into desired chemicals in high yields avoiding the syngas route, as reviewed by Tang et al. [3]. The ultimate goal is to better understand the fundamental interactions and mechanisms underlying the key catalytic processes in order to develop cost-efficient strategies for methane (and other hydrocarbons) activation. To this end, a wide variety of fundamental experimental and theoretical studies have investigated the reactivity of bare transition metal monocations with methane [7–56]. These methods have been extensively reviewed and highlight the varied conditions and energetic requirements that yield particular products upon reaction [57–62].

The metal-catalysed dehydrogenation of methane (Reaction 1) is one reaction of key interest. This reaction is thermodynamically-feasible (i.e., exothermic) if the metal–methylidene bond dissociation energy exceeds the heat of dehydrogenation of methane (∆_r_ *H* = 464 kJ mol^−1^ [35, 60, 61]). This reaction often begins with the formation of an M^+^(CH_4_) adduct followed by C–H bond insertion via oxidative addition to yield a hydridomethyl intermediate (H–M^+^–CH_3_). From here, H–H bond coupling and subsequent H_2_ elimination occurs via α-hydrogen transfer to yield a metal–methylidene product (MCH_2_^+^) [40, 59, 61].

1 $${M^+}+C{H_4} \to MCH_{2}^{+}+{H_2}$$

Of the ground-state transition metal ions investigated, 5*d* transition metal cations (specifically, Ta^+^, W^+^, Os^+^, Ir^+^, and Pt^+^) dehydrogenate methane the most efficiently [13, 15, 16, 35]. Ground-state 3*d* and 4*d* transition metals (with the notable exception of Nb^+^ [35]) rather than dehydrogenating methane, tend to undergo CH_4_ addition reactions leading to the formation of M^+^(CH_4_)_*n*_ ion–molecule complexes [7, 35], often without substantial C–H bond activation. Unlike the ground state, electronically-excited Cr^+^ does dehydrogenate methane exothermically [55] as does ground-state Ti^+^ in coordination to multiple methane ligands [41]. In the latter case, complexation reduces the barrier to ion insertion into the C–H bond, thus favouring the formation of the key hydridomethyl intermediate in Ti^+^(CH_4_)_*n*_ (*n* ≥ 3) complexes [41]. Extensive guided ion beam experiments have also shown that Reaction 1 occurs for various 3*d* and 4*d* transition metal monocations under single collision conditions at high collision energies. Modelling of the endothermic cross-sections can then provide metal–hydrogen and metal–carbon bond dissociation energies [8–12, 14, 18, 19, 23, 24, 26, 28, 29, 63].

Despite the extensive study of methane dehydrogenation, investigations focussing on the M^+^(CH_4_) adduct remain scarce. In this adduct, the bonding is believed to involve a combination of electrostatic and covalent interactions, with the hapticity controlled by the dominant interaction. For example, in Cu^+^(CH_4_), electrostatics favour η^3^ coordination whilst covalent interactions favour η^2^ coordination—[64]. The covalent interaction can generally be described with a simple donor–acceptor model [61] in which a σ-complex is formed involving the donation from C–H σ orbitals into (partially) empty orbitals on the metal, with concomitant back-donation from occupied metal π–orbitals into antibonding C–H orbitals. In some cases, this model has been expanded to include the effects of *s*–*d* hybridization, which often leads to a reduction of repulsion between the M^+^ and CH_4_ along the axis of interaction and an increased electron donation from proximate C–H σ bonds into vacant *s–d* hybridized orbitals [50, 56]. Overall, the M^+^–CH_4_ interaction leads to a red-shift in the stretching vibrations associated with proximate C–H bonds, the magnitude of which is sensitive to the electronic configuration (including the influence of promotion energies), orbital sizes, and exchange energy [16]. For M^+^(CH_4_)_*n*_ complexes, the number and coordination environment of each ligand also influence the extent of C–H activation.

Overall, the red-shift in the stretching vibrations of the C–H bonds of CH_4_ upon metal complexation makes photofragmentation spectroscopy an ideal technique to probe the geometry of M^+^(CH_4_)_*n*_ (*n* ≥ 1) complexes and hence the degree of C–H activation [65–67]. The *s*-block metal complexes Mg^+^(CH_4_) [68] and Ca^+^(CH_4_) [69], and transition metal complexes V^+^(CH_4_) [70] and Zn^+^(CH_4_) [71], have been studied via UV/Visible photofragmentation action spectroscopy in the vicinity of atomic metal ion resonances. Previous IR spectroscopic studies of M^+^(CH_4_)_*n*_ complexes include those of *s*-block and *p*-block metals Li^+^(CH_4_)_1−6_ [72, 73] and Al^+^(CH_4_)_1−6_ [74], respectively. These complexes were found to be dominated by electrostatic interactions due to the inert electronic configurations of Li^+^ and Al^+^, with high charge density and a small ionic radius also contributing to the former. No measurable effects of ligand–ligand interactions in the first and second coordination shells were observed for these complexes. These electrostatic interactions were found to weaken as the number of ligands increases (characterised by a smaller red-shift), and as competition for interaction with the metal ion increases.

First-row transition metal complexes such as Mn^+^(CH_4_)_1−6_ [75], Fe^+^(CH_4_)_1−4_ [76], Co^+^(CH_4_)_1−4_ and Ni^+^(CH_4_)_1−4_ [77] have also been investigated via IR photofragmentation spectroscopy [75]. The Fe^+^, Co^+^ and Ni^+^ ions, with their 3*d* ^*n*^ ground state configurations, interact with methane more strongly than ions with 3*d* ^*n*−1^4*s* ^1^ configurations, such as Mn^+^(CH_4_)_1−6_. IR photofragmentation spectroscopy studies have also been performed on the *d* ^10^ complexes Cu^+^(CH_4_)_1−6_ and Ag^+^(CH_4_)_1−6_ [78], which tend to possess highly-symmetrical structures due to the spherical nature of the ion. The latter studies inspired the work presented here as part of our own development of a metal–ligand complex infrared dissociation instrument.

Despite all of the photofragmentation spectroscopy investigations on numerous M^+^(CH_4_)_*n*_ systems performed previously, there remains a paucity of studies involving 5*d* transition metal ions. Of these, Au^+^ is of particular interest owing to its prolific use in homogenous catalysis [79–109], the emergence of which has been motivated by the unique properties of Au that are governed by strong relativistic effects [110–115]. Previous work on Au^+^(CH_4_)_*n*_ complexes (*n* ≥ 1) includes an ICP/SIFT study involving the reactivity of Au^+^ with CH_4_ by Bohme and co-workers [35]. They observed a low propensity for formation of high-order Au^+^(CH_4_)_*n*_ complexes with no dehydrogenation products observed. A combined guided ion beam/theoretical study by Li and Armentrout [33] observed a unique mechanism for the dehydrogenation of methane by Au^+^ which occurs without the involvement of an oxidative addition intermediate. Instead, AuCH_2_^+^ and H_2_ apparently form directly from the Au^+^(CH_4_) adduct without the involvement of a transition state, making this mechanism unique among the 5*d* transition metals.

Here, we report the results of an IR-REPD spectroscopic investigation of gas-phase Au^+^(CH_4_)_*n*_ complexes (*n* = 3–8), employing the inert messenger or “rare-gas tagging” technique, whereby loss of a weakly-bound argon atom provides a mass spectrometric signature of IR photon absorption. This technique has previously been utilized by our group in the IR-REPD studies of M^+^(CO_2_)_*n*_ complexes (M = Co, Rh, Ir) [116], M^+^(N_2_O)_*n*_ complexes (M = Cu, Ag, Au) [117] and IR-MPD studies of larger bare and decorated transition metal clusters [118–124]. In separate experiments, we have observed enhanced dehydrogenation when methane is vibrationally excited prior to interaction with Au^+^.

## Experimental and Computational Details

The instrument used in these studies has been described previously [116] and only basic details are given here. Gold ions are produced by pulsed laser ablation of a rotating gold disk target using 5 mJ of 532 nm light from a frequency doubled Nd:YAG laser (Continuum Minilite, 8 ns pulse). For rare-gas tagging, the ions are entrained in a pulse of He carrier gas, seeded with 2% methane and 20% argon, delivered by a solenoid valve (Parker-Hannifin, Series 9).

IR photodissociation of the argon-tagged complexes is performed using tunable IR light, generated from an OPO/OPA laser scanned in the region of the *a*_1_ symmetric stretch (2917 cm^−1^) and *t*_2_ stretch (3019 cm^−1^) of free CH_4_. Whenever the incident IR light is resonant with an IR-active mode of an Au^+^(CH_4_)_*n*_Ar complex, photons can be absorbed and, following intramolecular vibrational redistribution (IVR), the weakly-bound Ar atom is lost providing a spectral signature of the photon absorption. IR-REPD spectra are generated by monitoring the efficiency of Ar loss from the parent complex, as a function of wavelength.

To aid in the interpretation of the experimental spectra, we have performed spectral simulations of low-energy isomers using density functional theory. A range of candidate starting structures for Au^+^(CH_4_)_*n*_Ar and Au^+^(CH_4_)_*n*_ were optimized using the B3P86 density functional [125, 126] coupled with the Def2TZVP basis set [127, 128]. Using the Def2TZVP basis set allows for 60 core electrons of Au to be treated with a quasirelativistic *ab initio* pseudopotential developed by Andrae et al. [129], which has been used successfully by our group to rationalize binding trends observed for M^+^(N_2_O)_*n*_ complexes (where M = Cu, Ag, Au) [117]. From harmonic vibrational frequency calculations of the structures obtained, IR spectra were simulated by convoluting the line spectra with a Lorentzian line shape to match the experimental resolution. The simulated IR spectra were scaled for comparison with experimental IR-REPD spectra. The scaling factor (0.95538) was derived from comparison of the calculated 3160 cm^−1^ *t*_2_ stretching frequency of CH_4_ with the experimental value of 3019 cm^−1^. Calculations were performed both with and without Ar atoms in order to determine any effects of the rare gas tag. All calculations were performed using the Gaussian09 suite of programs [130].

## Results and Discussion

**Fig. 1:**
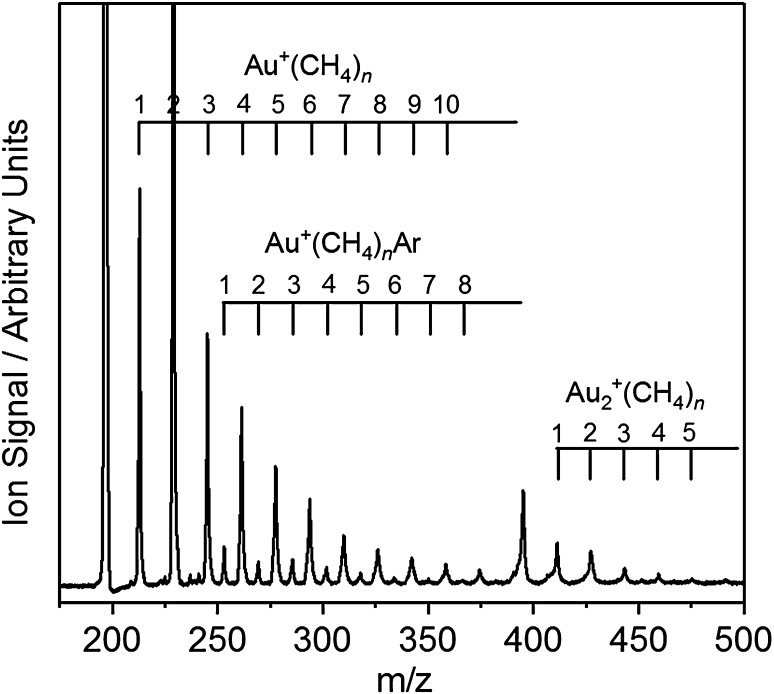
Time-of-flight mass spectra of Au^+^(CH_4_)_*n*_, Au_2_^+^(CH_4_)_*n*_, and Au^+^(CH_4_)_*n*_Ar complexes produced upon laser ablation of a gold target in the presence of a 2% CH_4_/20% Ar in He gas mixture at 6 bar backing pressure. The Au^+^(CH_4_)_2_ complex is produced with the highest signal intensity, reflecting the stability of this species

Figure 1 shows a typical time-of-flight mass spectrum obtained by laser ablation of a gold target in the presence of 2% CH_4_/20% Ar in He gas mixture at a backing pressure of 6 bar. The range of clusters generated can be tuned crudely with a combination of backing gas composition and backing pressure. A range of Au^+^(CH_4_)_*n*_, Au_2_^+^(CH_4_)_*n*_ and Au^+^(CH_4_)_*n*_Ar complexes can be assigned as shown. The Au^+^(CH_4_)_2_ complexes are produced with the strongest intensities—an early indication of the particular stability of this coordination number. In the absence of Ar tagging, and/or at higher CH_4_ partial pressures, the *n* = 2 complex is also the most intense for the Au_2_^+^ complexes.

### IR Depletion Spectra of Au^+^(CH_4_)_*n*_Ar Complexes

Under the experimental conditions employed here, Ar-tagging is successful for Au^+^(CH_4_)_*n*_ up to *n* = 8 generating signals up to 10% of the corresponding non-tagged peaks. IR depletion spectra were obtained with acceptable signal-to-noise for the *n* = 3–8 complexes.

**Fig. 2:**
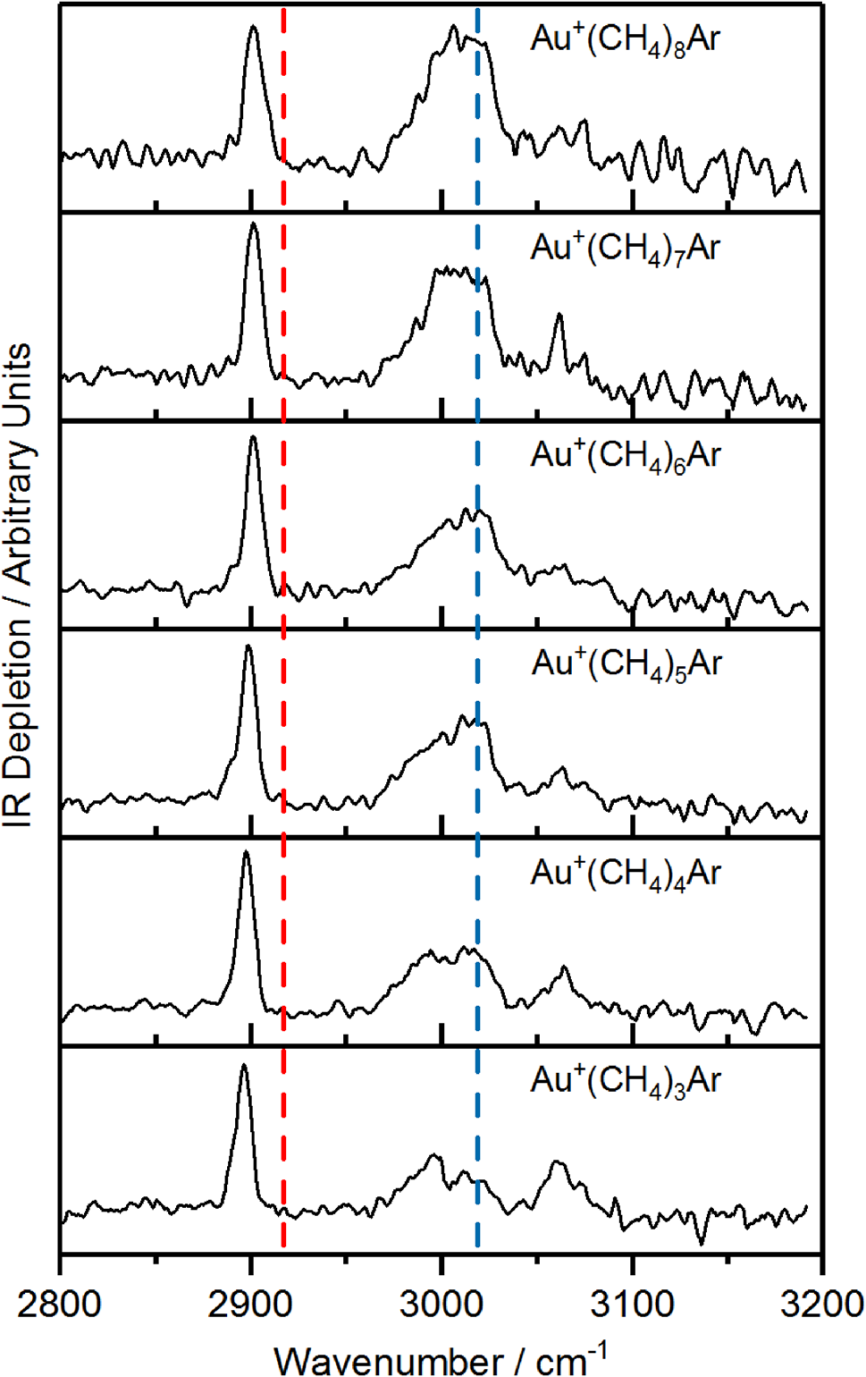
IR-REPD spectra of argon-tagged Au^+^(CH_4_)_*n*_ complexes (*n* = 3–8) recorded between 2800 and 3200 cm^−1^. The dashed red line at 2917 cm^−1^ and dashed blue line at 3019 cm^−1^ indicate the positions of the fundamental bands of the *a*_1_ symmetric stretch and *t*_2_ stretch of free CH_4_, respectively

Figure 2 shows the IR depletion spectra recorded for the Au^+^(CH_4_)_*n*_Ar (*n* = 3–8) complexes in the region 2800–3200 cm^−1^ which encompasses the fundamental transitions of the *t*_2_ and *a*_1_ normal modes in free CH_4_. All spectra in this range are very similar each displaying three notable features: (i) A narrow band appearing at *ca*. 2900 cm^−1^, (ii) a broad, largely unresolved feature at *ca*. 3000 cm^−1^, and (iii) a weaker feature appearing at *ca*. 3065 cm^−1^.

The narrow intense band at *ca*. 2900 cm^−1^ can be assigned to a totally-symmetric *a*_*1*_ C–H stretch in CH_4_ that becomes IR active upon complexation with Au^+^. This band is only slightly red-shifted relative to the *a*_*1*_ fundamental in free CH_4_ (red-dashed line in Fig. 2, *ca*. 2917 cm^−1^ [131]). As the number of CH_4_ ligands (*n*) increases, this band blue-shifts slightly by *ca*. 3 cm^−1^ as the interaction of the Au^+^ with an increasing number of ligands diminishes the perturbation on individual CH_4_ moieties. The other features in the IR spectra are related to the methane *t*_*2*_ stretch (whose wavenumber in free CH_4_, *ca*. 3019 cm^−1^, is marked by a blue-dashed line in Fig. 2 [132]).

Qualitatively similar features within the same IR range have also been observed in the IR depletion spectra of the other *d* ^10^ metal cation complexes, Cu^+^(CH_4_)_4−6_ and Ag^+^(CH_4_)_5−6_ recorded previously by Metz et al. [78]. However, for *n* = 3 the IR depletion spectra presented here for the gold complexes differ significantly from those of the other coinage metals indicating a markedly different binding motif.

### DFT Calculations

#### Structural Dynamics and Band Analysis

**Fig. 3:**
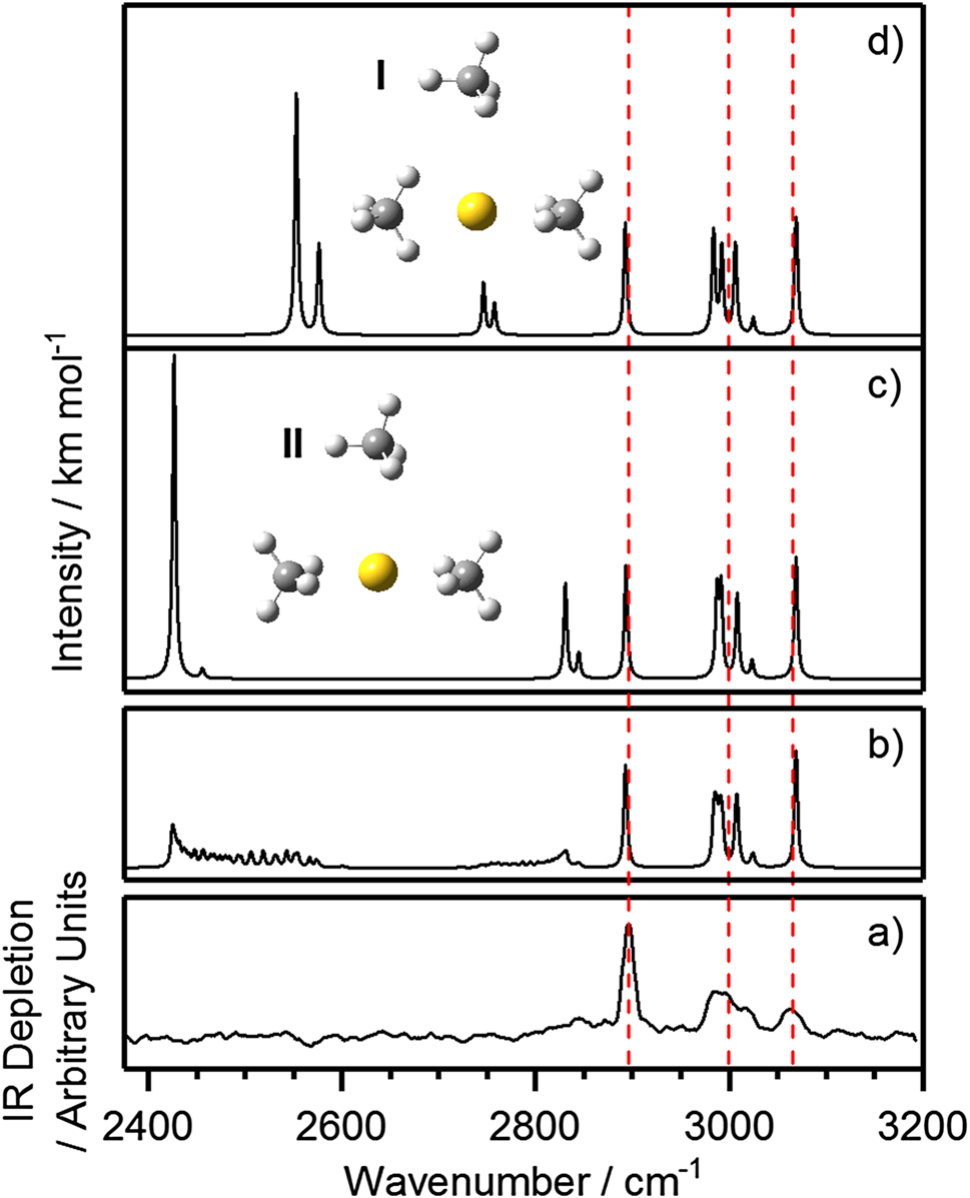
**a** Low-resolution IR-REPD spectrum of Au^+^(CH_4_)_3_Ar compared with simulated IR spectra of the: **c** eclipsed, and **d** staggered minima calculated at the UB3P86/Def2TZVP level of theory, with a scaling factor of 0.95538 applied to the simulated spectra. A simulated IR spectrum (**b**) generated by summing together individual IR spectra taken at various orientations between the two minima is also shown. Free internal rotation washes out the spectral features below 2850 cm^−1^ which arise from proximal C–H stretches in the core ligands. The dashed red lines indicate the positions of the peaks in the experimental IR spectrum. Calculations show that including the argon tag has minimal effect on the Au^+^(CH_4_)_3_ complex (and most likely Au^+^(CH_4_)_*n*,_ generally)

For the *n* = 3 complex, DFT calculations yield two near-equivalent minima of significance as shown in Fig. 3. Both comprise a linear Au^+^(CH_4_)_2_ core structure in which the methane ligands are *η* ^2^ coordinated to the Au^+^ center, with a third, more weakly-bound ligand in a T-shaped structure. The primary difference between the two minima is whether the proximal hydrogens in the core ligands are staggered (Structure I—Fig. 3) or eclipsed (Structure II—Fig. 3) when viewed along the C–Au–C axis. These two structures are essentially isoenergetic with DFT predicting structure I to lie higher in energy by *ca*. 1 meV.

**Fig. 4:**
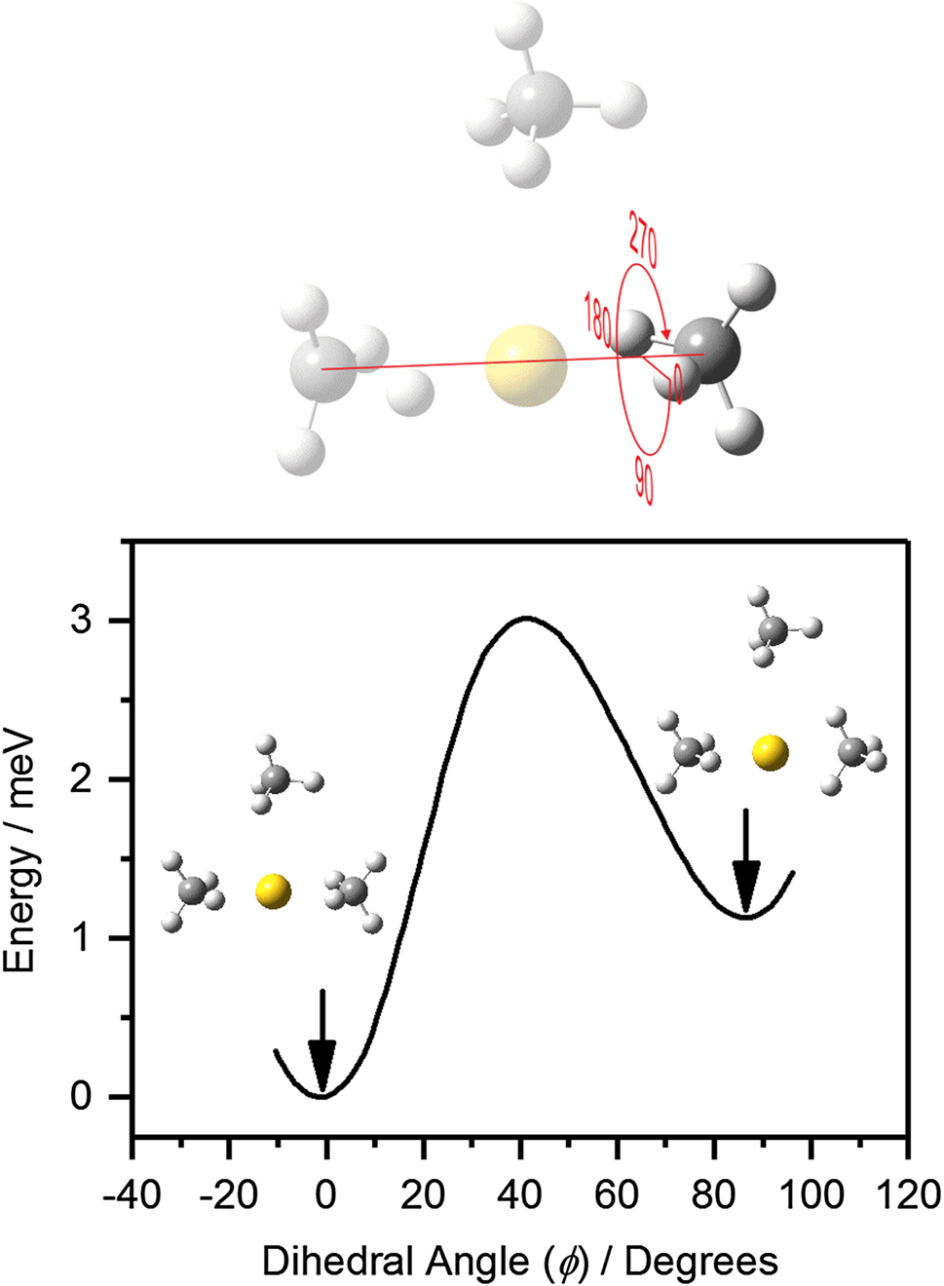
Relaxed scan calculated energy of Au^+^(CH_4_)_3_ as a function of dihedral angle (defined above) between two C–H bonds on both core methane ligands for a rotation of 90°. The eclipsed structure is marginally the lower energy of the two minima with a small barrier to internal rotation

Comparison of the IR-depletion spectrum for Au^+^(CH_4_)_3_Ar with the simulated IR spectra of both structures reveal interesting insights into the structural dynamics of this complex (Fig. 3c, d). The spectra of the two structures are nearly identical in the range 2850–3200 cm^−1^ with predicted features clearly recognizable in the experimental spectrum. However, strong additional bands—different for each structure—are observed in spectra simulated in the range 2400–2850 cm^−1^, where the experimental spectrum shows no such features. It is clear from the simulations that the wavenumbers of these lower-energy bands depend strongly upon the relative dihedral angle between the two core methane ligands. A relaxed scan of this dihedral angle between the eclipsed and staggered minima (Fig. 4) reveals a negligible 3 meV barrier to internal rotation, implying free rotation of the two inner-shell methane molecules. The effect of such motion is to wash out the spectral features in the 2400–2850 cm^−1^ region from the IR-REPD spectrum leaving only the persistent bands > 2850 cm^−1^ (see Fig. 3b) to be observed in the spectrum.

**Fig. 5:**
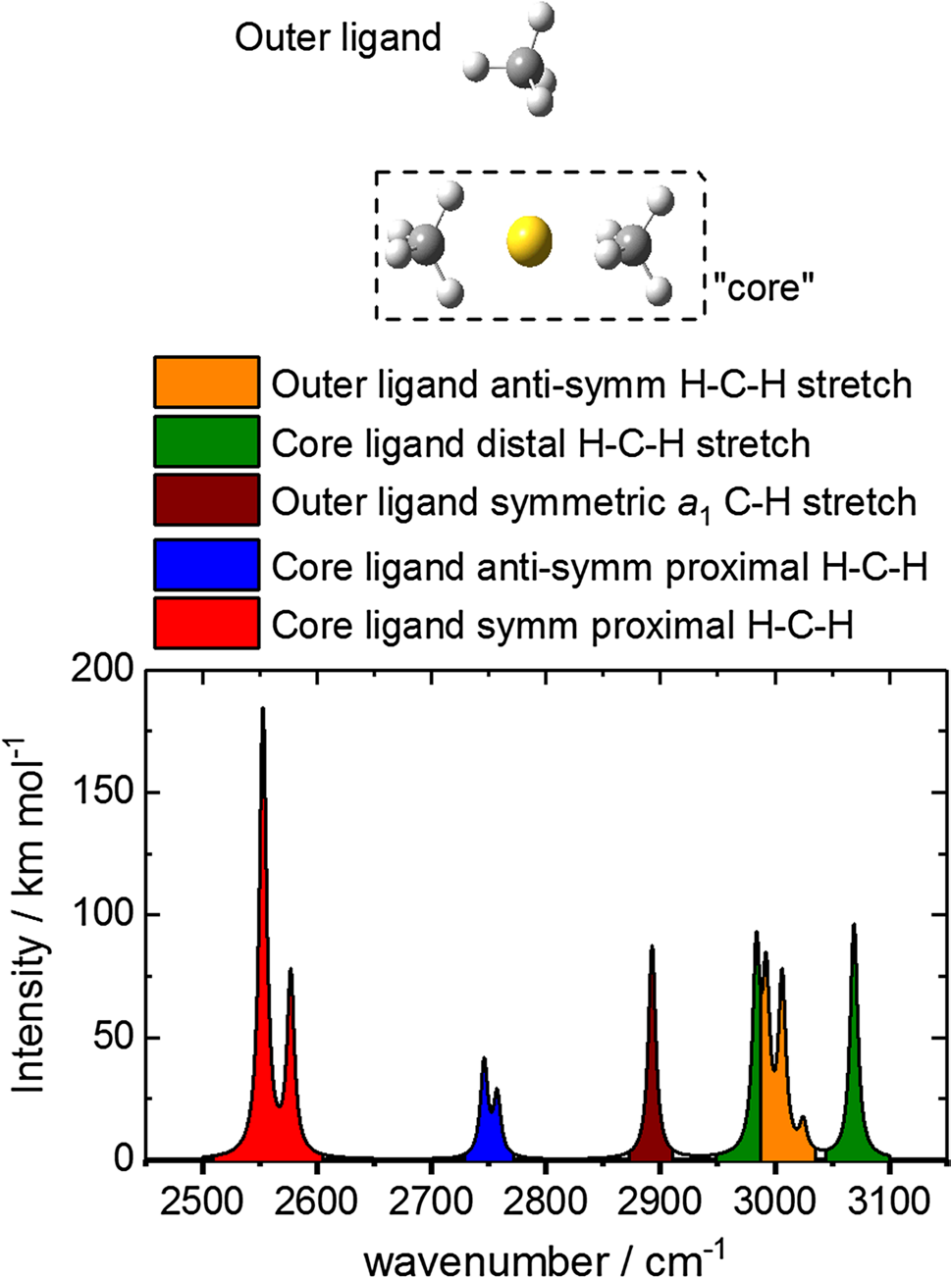
Structure of the Au^+^(CH_4_)_3_ complex with staggered core structure and its assigned simulated IR spectrum in the region 2500–3100 cm^−1^

Further confirmation of the effects on the IR spectrum of free rotation of the core ligands is provided by the interpretation of the bands in the simulated IR spectrum of Structure I, shown in Fig. 5. The ten predicted bands in the IR spectrum can be grouped into five distinct groups based on the molecular motions involved.

The two bands at *ca*. 2550 cm^−1^ arise from combinations of symmetric proximal H–C–H stretches of the two core methane ligands. The second group—the two weaker bands at *ca*. 2750 cm^−1^—involves the anti-symmetric proximal H–C–H stretches of the same ligands. All of these bands are exquisitely sensitive to the dihedral angle and their wavenumber and intensity change continuously with the relative angle of the two ligands.

The rest of the bands observed in the IR-REPD spectrum of the *n* = 3 complex arise from motion either involving the outer ligand or distal C–H stretches of the core ligands. The 2900 cm^−1^ band can be confidently assigned due to a fully-symmetric (*a*_1_) C–H stretch in the third, more weakly–bound ligand. The broad feature observed experimentally at *ca*. 3000 cm^−1^ has contributions from different *t*_2_-type stretches including symmetric distal H–C–H stretches of the inner-core methane ligands, and three *t*_2_ modes of the weakly-bound ligand (whose degeneracy is lifted by the interaction with Au^+^). Finally, the feature at 3065 cm^−1^ arises from the anti-symmetric distal H–C–H stretches (also of *t*_2_ symmetry relative to free CH_4_) of the core ligands. These same three groups also appear in the simulated spectrum of the eclipsed structure (Structure II—Fig. 3), confirming their persistence during internal rotation of the core ligands.

The binding of the two core ligands in a *η* ^2^ fashion suggests that these interactions have significant covalent character. This is consistent with the calculations of Maitre and Bauschlicher on Cu^+^(CH_4_) [64], in which *η* ^2^ coordination to the Cu center arises due to covalent interactions whereas *η* ^3^ coordination, by contrast, arises from electrostatic interactions. Metz and co-workers [78] offer more specific details of the covalent interaction, concluding that *η* ^2^ coordination involves two charge transfer processes: (i) σ-donation from the Cu 3*d*_*z*_^2^ orbital to empty anti-bonding ligand orbitals, and (ii) σ-back-donation from the CH_4_ *t*_2_ bonding orbitals into the empty Cu 4*s* orbital. Equivalent interactions are likely to govern the interactions in the Au^+^(CH_4_)_2_ core.

In an attempt to simulate the likely effect on the spectrum of the free internal rotation, we have convoluted the spectra calculated for structures at every 5° of dihedral angle between 0 and 90 (see Fig. 3 b). As expected, only the spectral features arising from the distal core ligands and those associated with the outer coordination shell ligands persist with significant intensity and the spectrum provides a satisfactory fit to the experimental data.

For the rest of the Au^+^(CH_4_)_*n*_Ar complexes (*n* = 4–8) there is good agreement between the experimental IR-REPD spectra and the simulated spectra of their putative global minimum structures. This is to be expected as each calculated structure comprises the same linear Au^+^(CH_4_)_2_ core (with mean Au^+^–CH_4_ bond lengths of *ca*. 2.3 Å) to which additional methane ligands bind equatorially up to *n* = 6 (with average Au^+^–CH_4_ bond lengths of *ca*. 3.5 Å) until a second coordination shell is complete. Beyond *n* = 6, additional methane molecules bind at even larger Au^+^–CH_4_ bond lengths (*ca*. 5.8 Å).

Since the IR spectrum is dominated by features in the common core only minor changes are observed with increasing ligand number. The assignments made for the *n* = 3 complex apply to all complexes, with visualization of the numerous modes for all global minima confirming this assertion. The slight blue-shift and broadening of the experimental band at *ca*. 2900 cm^−1^ as *n* increases is due to an increasing number of outer ligands contributing to this particular feature. Likewise, the increasing number of outer ligands accounts for the increase in relative intensity of the broad 3000 cm^−1^ feature.

#### Trends in Binding Energy and Au^+^–C Bond Distances

**Fig. 6:**
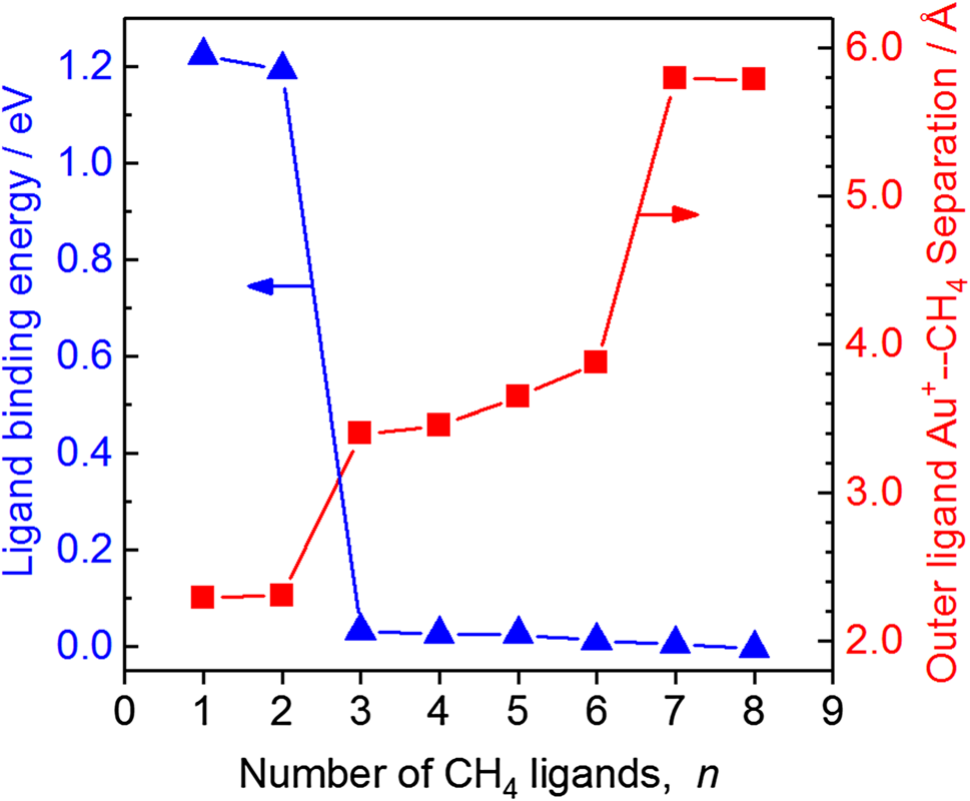
The greatest Au–C internuclear separation (red, right hand scale) and binding energy of the *n*th methane (blue, left hand scale) in Au^+^(CH_4_)_*n*_ (*n* = 1–8) plotted against the number of methane ligands, *n*. The step-changes between *n* = 2 and 3, and between *n* = 6 and 7 indicate the existence of a core structure with two methane ligands and a second-shell of four methane ligands

Further evidence for an inner-core of two methane ligands in Au^+^(CH_4_)_*n*_Ar complexes is provided by both: (i) the DFT-calculated binding energy of CH_4_ ligands, and (ii) the longest Au^+^–C internuclear separation in each complex as a function of ligand number (see Fig. 6). The first two ligands to bind do so with large binding energies (*ca*. 1.2 eV). This is a factor two larger than the equivalent binding energies calculated by Kocak et al. for Ag^+^(CH_4_)_1,2_ and larger even than for Cu^+^(CH_4_)_1,2_ [78]. By contrast, the third ligand has a binding energy more than an order of magnitude smaller.

The same effect is mirrored in the trends in Au^+^–C internuclear separation. The inner, core ligands have mean metal–ligand separations of 2.3 Å but the third ligand binds at a mean distance of 3.5 Å away. A second step in this trend is observed between *n* = 6 and 7 as the second solvation shell closes, the 7th ligand is a distant 5.8 Å from the metal centre.

The observation of a linear Au^+^(CH_4_)_2_ core structure in the complexes studied here is not surprising—Au^+^–L_2_ structures are common. As first postulated by Orgel [133], this behavior is often attributed to hybridization of the 6*s* and 5*d*_*z*_^2^ orbitals of Au^+^—which is very efficient due to relativistic effects. These *s*–*d*_*z*_ hybrid orbitals favor the linear interaction of two ligands on opposing sides of the Au^+^ center.

### Reactions of Au^+^/Au^+^(CH_4_)_*n*_ with Vibrationally-Excited CH_4_

The IR-REPD experiments above rely on first forming metal–ligand complexes and then interrogating them downstream in the molecular beam with the IR pulse. We have also performed a conceptually different series of experiments in which the IR pulse is fired just *before* the metal ablation laser. This is made possible by the counter-propagating nature of the IR laser and the molecular beam in our instrument which allows the IR laser to penetrate through the skimmer orifice right up to the cluster source.

**Fig. 7:**
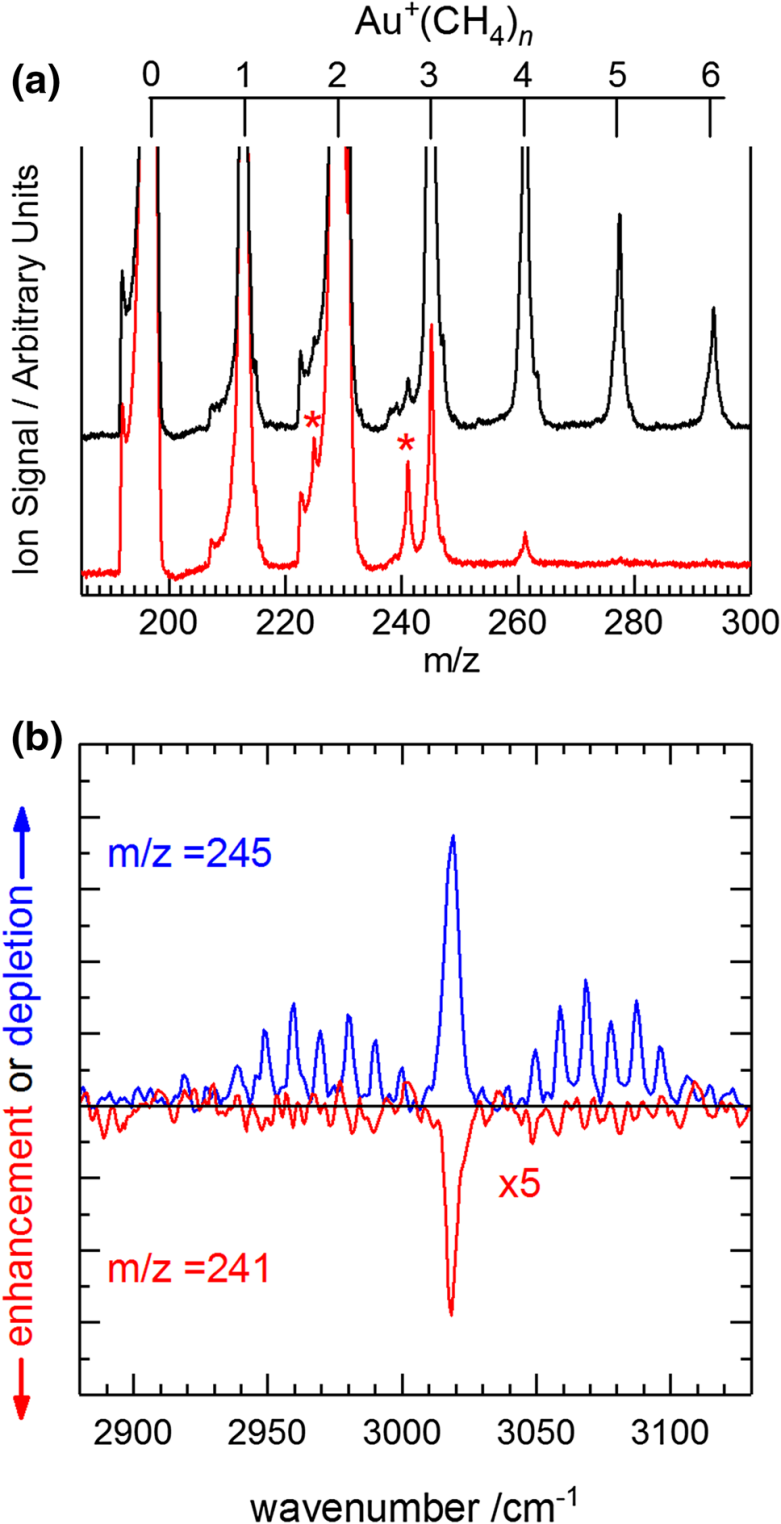
**a** Mass spectra of Au^+^(CH_4_)_*n*_ complexes without (black) and with (red) the IR laser pulse, resonant on the *Q*-branch in the CH_4_ *t*_*2*_ fundamental band at 3019 cm^−1^, fired immediately before the ablation laser pulse. The asterisks indicate observed enhancement in signals at m/z = 225 and 241 assigned to dehydrogenation products arising from reactions with vibrationally excited CH_4_. **b** The corresponding IR spectrum of the depletion signal on Au^+^(CH_4_)_3_ (blue) indicating reduced complexation efficiency upon ligand IR excitation and enhancement (red) of the Au^+^[C_3_, H_8_] signal at m/z = 241

In these experiments only the IR active, *t*_2_ stretching mode of free methane at 3019 cm^−1^ is excited. As shown in Fig. 7, vibrationally-exciting the methane in the beam has two significant effects: (i) it results in a *reduction* in the generation of Au^+^(CH_4_)_*n*_ complexes (particularly *n* > 2), and (ii) it leads to an *enhancement* in minor peaks in the mass spectrum 4u lower than the main Au^+^(CH_4_)_*n*_ complexes’ peaks.

The reduction in the efficiency of complexation following vibrational excitation of the ligand is unsurprising—this represents additional internal energy which must be lost in subsequent cooling collisions for the complex to remain bound. Furthermore, IR excitation of CH_4_ in the comparatively high pressure region of the cluster source most likely produces a photoacoustic signal and shock-wave which heats the beam locally. When the IR laser is resonant with the *Q*-branch of the *t*_2_ fundamental, the signal of the *n* = 3 complex is reduced by 80% and is accompanied by almost complete loss of the larger complexes (see Fig. 7a).

The reduction in the efficiency of generating Au^+^(CH_4_)_*n*_ complexes resulting from CH_4_ vibrational excitation is sufficiently strong to permit a spectrum of this “complex inhibition” to be recorded (Fig. 7b), recorded in depletion of the Au^+^(CH_4_)_3_ signal as a function of IR wavenumber. This spectrum reflects well the IR absorption spectrum of CH_4_ of the *t*_2_ fundamental band with preliminary modelling of the spectral band contours suggesting a slight cooling within the short cluster channel following expansion from the valve.

The second, more interesting, effect observed in Fig. 7 is a marked enhancement in the intensity of peaks in the mass spectrum corresponding to dehydrogenation products following reaction with vibrationally-excited methane molecules. This is particularly noticeable at m/z = 225 (corresponding to Au^+^(CH_4_)_2_–4u) and 241 (Au^+^(CH_4_)_3_–4u), respectively). In the same way that the CH_4_ IR absorption spectrum is reproduced in the depletion of Au^+^(CH_4_)_*n*_, the same spectrum is manifest in the absolute *enhancement* of the signal observed at m/z = 241 (see Fig. 7b), confirming the role of the infrared excitation of CH_4_ in the production of this species.

Clearly these absolute enhancements imply an increase in reactivity as a result of IR excitation of the CH_4_ though we can only speculate as to: (i) the reaction(s) which is(are) enhanced (CH_4_* + Au^+^ or CH_4_* + Au^+^(CH_4_)_*n*_), and (ii) the nature of the reaction products since we have no explicit spectral information beyond the mass of the species produced. In this context, it is worth noting that, in their recent mid-IRMPD study of the results of Pt^+^ + CH_4_ reactions/clustering, Bakker, Armentrout and co-workers observed significant production of Pt^+^(CH_3_)_2_(CH_4_)_*n*_ from collisions during the clustering processes [134]. This product was interpreted as arising from methane C–H activation by Pt^+^ generating Pt^+^CH_2_ which subsequently reacts with additional methane to produce the di-methyl complexes. In a wider context, Beck and coworkers have studied vibrationally-mediated dissociative chemisorption of CH_4_ isotopologues on extended transition-metal surfaces observing mode-specific dissociation [135–137].

More generally, single dehydrogenation of methane (i.e., H_2_ loss) within M^+^(CH_4_)_*n*_ complexes (M = Nb and Ta; *n* = 1–4) has been investigated theoretically previously, and two common reaction mechanisms—termed ‘direct activation’ and ‘cluster-assisted activation’—are believed to form two different products [47]. The ‘direct activation’ mechanism involves oxidative insertion of M^+^ into a C–H bond followed by H migration leading to formation of the (H_2_)–M^+^–(CH_2_) intermediate, followed by reductive elimination of H_2_ (Scheme 1):

2 $$\begin{aligned} {\text{M}}^{ + } \left( {{\text{CH}}_{4} } \right) & \to \,{\text{H}} - {\text{M}}^{ + } \left( {{\text{CH}}_{3} } \right) \to {\text{insertion}} \\ & \to {\text{H}}_{2} - {\text{M}}^{ + } \left( {{\text{CH}}_{2} } \right) \to {\text{H-migration}} \\ &\to {\text{M}}^{ + } \left( {{\text{CH}}_{2} } \right) + {\text{H}}_{2} \to {\text{H}}_{2} {\text{loss}} \\ \end{aligned}$$

Under single collision conditions, Irikura and Beauchamp found several third-row transition metal ions, namely W^+^, Ta^+^, Os^+^, Ir^+^ and Pt^+^ to dehydrogenate methane consistent with this mechanism. Au^+^, however, was found to be unreactive with methane under ion cyclotron resonance conditions but did slowly dehydrogenate ethane [15, 16].

More recently, in guided ion beam studies, Li and Armentrout found that, in the case of Au^+^ + CH_4_, due to the 5*d* ^10^ ion configuration the barrier for the oxidative addition/reductive elimination reaction is much too high to account for the reaction efficiencies they observed at low collision energies. Instead dehydrogenation occurs, endothermically, directly from the Au^+^(CH_4_) adduct without an intermediate transition state [33].

Within *n* > 2 complexes, in addition to the mechanism outlined in Scheme 1 which generates species of the form M^+^(CH_2_)(CH_4_)_*m*_, a ‘cluster-assisted’ mechanism leading to the formation of another product is also plausible whereby insertion into a C–H bond is followed by H migration from a different methane ligand followed by H_2_ loss to leave the dimethyl complex (Scheme 2):

3 $$\begin{aligned} {\text{M}}^{ + } \left( {{\text{CH}}_{4} } \right)_{n} & \to \,{\text{H}} - {\text{M}}^{ + } \left( {{\text{CH}}_{3} } \right)\left( {{\text{CH}}_{4} } \right)_{{n - 1}} \to {\text{insertion}} \\ &\to {\text{H}}_{2} - {\text{M}}^{ + } \left( {{\text{CH}}_{3} } \right)_{2} \left( {{\text{CH}}_{4} } \right)_{{n - 2}} \to {\text{H-migration}} \\ &\to {\text{M}}^{ + } \left( {{\text{CH}}_{3} } \right)_{2} \left( {{\text{CH}}_{4} } \right)_{{n - 2}} + {\text{H}}_{2} \to {\text{H}}_{2} {\text{loss}} \\ \end{aligned}$$

In our study, only double-dehydrogenation (i.e., loss of two units of H_2_) is observed clearly, resulting in enhancements of the Au^+^[C_2_, H_4_] and Au^+^[C_3_, H_8_] species. We cannot exclude the possibility of some H_2_ loss with our mass resolution, but there is no obvious peak in Fig. 7 at m/z 243. Nevertheless, it is possible to conceive extended versions of the above which result in the loss of two H_2_ molecules. Clearly, reaction pathway calculations are required which would be informed by spectroscopy of the reaction products. This would require post-reaction spectroscopic studies of the type performed by Wheeler et al. [134] and the lack of availability of multiple IR lasers prevented such studies here.

## Conclusions

Infrared spectroscopy combined with spectral simulations based on density functional theory have revealed a linear core Au^+^(CH_4_)_2_ structure to small gas-phase Au^+^(CH_4_)_*n*_ metal–ligand complexes. The core ligands bind in a *η* ^2^ motif with evidence of incipient chemical bonding with additional ligands binding closer to *η* ^3^. In the region of the CH_4_ *a*_1_ and *t*_2_ vibrational modes, free internal rotation of the core washes out several spectral features leaving only persistent bands based on distal CH_2_ stretches and vibrations in non-core ligands.

Vibrational excitation of methane before interactions with metal atoms/ions are possible, leads to an expected reduction in the number density of complexes produced but also an enhancement in (double–) dehydrogenation products. This latter observation, arising presumably from enhanced C–H activation in the vibrationally-excited ligand, offers the possibility of mode-selective intra-cluster chemistry similar to that observed in dissociative chemisorption of methane on extended transition metal surfaces.
